# Calculations of relative intensities of fragment ions in the MSMS spectra of a doubly charged penta-peptide

**DOI:** 10.1186/1471-2105-13-S15-S13

**Published:** 2012-09-11

**Authors:** Tibor Pechan, Steven R Gwaltney

**Affiliations:** 1 Institute for Genomics, Biocomputing and Biotechnology, Mississippi Agricultural and Forestry Experiment Station, High Performance Computing Collaboratory, Mississippi State University, Mississippi State, MS 39762, USA; 2 Department of Chemistry, Center for Environmental Health Sciences, HPC2 Center for Computational Sciences, Mississippi State University, Mississippi State, MS 39762, USA

## Abstract

**Background:**

Currently, the tandem mass spectrometry (MSMS) of peptides is a dominant technique used to identify peptides and consequently proteins. The peptide fragmentation inside the mass analyzer typically offers a spectrum containing several different groups of ions. The mass to charge (*m/z*) values of these ions can be exactly calculated following simple rules based on the possible peptide fragmentation reactions. But the (relative) intensities of the particular ions cannot be simply predicted from the amino-acid sequence of the peptide. This study presents initial work towards developing a theoretical fundamental approach to ion intensity elucidation by utilizing quantum mechanical computations.

**Methods:**

MSMS spectra of the doubly charged GAVLK peptide were collected on electrospray ion trap mass spectrometers using low energy modes of fragmentation. Density functional theory (DFT) calculations were performed on the population of ion precursors to determine the fragment ion intensities corresponding to a Boltzmann distribution of the protonation of nitrogens in the peptide backbone amide bonds.

**Results:**

We were able to a) predict the *y *and *b *ions intensities order in concert with the experimental observation; b) predict relative intensities of *y *ions with errors not exceeding the experimental variation.

**Conclusions:**

These results suggest that the GAVLK peptide fragmentation process in the ion trap mass spectrometer is predominantly driven by the thermodynamic stability of the precursor ions formed upon ionization of the sample. The computational approach presented in this manuscript successfully calculated ion intensities in the mass spectra of this doubly charged tryptic peptide, based solely on its amino acid sequence. As such, this work indicates a potential of incorporating quantum mechanical calculations into mass spectrometry based algorithms for molecular identification.

## Background

Compared to empirical observation and statistical evaluation, the fundamental scrutiny and understanding of chemical processes pertinent to bio-systems represent qualitatively higher levels of knowledge. Seeking such knowledge, the experimental and theoretical approaches of chemistry must be applied in synergy to what have historically been considered typical biology problems. In recent years the methods of mass spectrometry have enabled a complex molecular analysis of organisms ranging from bacteria to plants to humans. In general, molecule identification is based on observing *m/z *values in the measured spectra and matching these values to an appropriate database or processing them *de-novo *to reveal molecule identity. In terms of instrumentation and software development for high-throughput analysis, the greatest advance has been achieved in proteomics applications of mass spectrometry [1].

Generally, in proteome studies the proteins are first separated and then digested with trypsin or another amino acid sequence-specific protease, and the resulting mixture of peptides is subjected to further analysis. Currently, tandem mass spectrometry (MSMS) of peptides is the dominant technique used to identify peptides and consequently proteins [2]. Understanding the peptide fragmentation pathways plays a key role in one's ability to interpret product ion spectra [3]. The peptide fragmentation inside the mass analyzer offers a peptide-typical spectrum of several different kinds of ion groups [4]. The *m/z *values of these ions can be exactly calculated following simple rules based on the possible peptide fragmentation reactions [5]. But the (relative) intensities of the particular ions cannot be arithmetically predicted from the amino-acid sequence of the peptide. Fragmentation of the parent peptide is uneven product-wise, and in the case of doubly charged tryptic peptides, it is facilitated by protonation of peptidic bonds in the precursor population, which is heterogeneous with respect to the site of the charge [6]. The acidic conditions (0.1% formic acid) during electrospray ionization (ESI) allow for protonation of all available basic sites in the peptide molecules (N-terminal amine and the basic side chains of lysine, arginine, and histidine residues). Doubly charged peptides dominate the tryptic digests of proteins, because of the proteolytic activity of trypsin that cleaves the amide at the C-terminal side of each lysine or arginine residue [7]. So, in general the peptides produced by the digest have at least two basic sites - the N-terminus and the side chain of the C-terminal lysine or arginine residue. In the gas phase the proton associated with the strongly basic side chain amino group of the C-terminal lysine or arginine is fixed at this site, even in collisional activation of peptide fragmentation [8]. But in contrast, a proton on the less basic N-terminus may move by internal solvation to any of the peptidic bonds [8]. During the peptide fragmentation (MSMS), the activation energy is converted into vibrational energy that is released through the charge driven dissociation reaction [6].

Well-established algorithms (Mascot [9], SEQUEST [10], X!Tandem [11], OMSSA [12]) are extensively used to identify peptides and consequently proteins. But more and more they are being scrutinized concerning their accuracy and reliability. One of the principal shortcomings of current algorithms is that either they ignore the observed spectral peak intensities of peptide fragment ions, or they utilize simple empirical general rules to account for them [13]. This approach to spectra analysis is a consequence of a knowledge gap in regard to ability to calculate explicitly not only the expected ion masses, but also their intensities. This gap prevents users from exploiting the full information (ion masses and intensities) contained in the spectra.

While the qualitative information in the MSMS spectra (ions' *m/z *values) is essential for peptide identification, the ions' intensities have been found to be significant as well, as documented by a number of studies. Researchers have applied statistical approaches [14,13,16] and machine learning algorithms [17-19], utilizing the data from tens to hundreds of thousands of spectra. These approaches managed both to identify factors affecting the ion intensities and to increase confidence in peptide identification. However, they remained descriptive in nature.

An apparent theoretical approach to ion intensity prediction is the work of Zhang [20-24]. His model is based on kinetics equations for unimolecular reactions involving many competing pathways, and it adopts the mobile proton theory of fragmentation. Zhang's model has seven major assumptions, takes into account eleven fragmentation pathways, and utilizes 236 parameters deemed important. A training data set of known spectra was used, and parameters were optimized until a best match was obtained between the predicted spectra and the experimental spectra. This "free parameterization is done by an unorthodox fitting scheme, and therefore the statistical properties of this scheme are not obvious" [13]. A reader may wonder if, after such empirical use of parameters, the underlying theoretical model still plays a significant role.

When reviewing the literature, one should also be aware that the word "computational" is liberally used to indicate not only terminus-technicus computational chemistry calculations but also the utilization of computers (for statistical analysis, search algorithms, calculating relative protein abundance, etc...) in general as well.

Using theoretical chemistry computational methods to elucidate peptide fragmentation has been pioneered by a few research groups. Historically, studies of singly charged peptides of limited length [2-5 amino acids (aa)] led the way [25-32]. But the doubly charged peptides comprised of 7-15 aa are the ones of crucial interest, due to their dominant presence in the real life data sets of peptides identified via liquid chromatography coupled to MSMS. Recently, studies utilizing DFT quantum mechanical calculations on doubly charged 3-7 aa peptides were published [33-36]. These works amassed extensive insight into the fragmentation mechanism, specific effects of certain amino acids (e.g. proline), kinetics of individual pathways, appearance, energy and structures of particular ions (*a, b*), and unexpected phenomena of sequence-scrambling. However the studies did not aspire to establish a unifying theory and a practical way to predict ion intensities based on quantum mechanical calculations.

The work presented in this article represents an initial step towards developing a theory-based computational tool allowing for the two-dimensional prediction of mass spectral data for peptides. Here, we present an algorithm for the prediction of fragment ion intensities in the spectrum of a doubly charged peptide based *solely on its amino acid sequence*. These results challenge the prevailing acceptance of a kinetic model [20,37] and support our hypothesis that the peptide fragmentation process in the ion trap mass spectrometer is predominantly driven by the thermodynamic stability of the precursor ions formed upon ionization of the sample, prior to the fragmentation.

## Methods

The synthetic penta-peptide GAVLK (GenScript) was dissolved in 5% acetonitrile and 0.1% formic acid for a final concentration of 5 pmol.μl^-1^. MSMS spectra of doubly charged GAVLK were collected on nano-electrospray ion trap mass spectrometers (LCQ Deca XP Plus and LTQ-Orbitrap Velos; THERMO), by direct sample injection (flow 500 nl.min^-1^), using low energy modes of fragmentation (CID-Collision Induced Decay, PQD-Pulsed-Q Dissociation). A combination of these mass detectors was used for the measurements: ion trap, linear ion trap, and orbitrap. Spectra were accumulated for five minutes via selected reaction monitoring (*m/z *= 244.3) and saved as individual files. The relative intensities of the *a-, b-*, and *y-*series of ions were quantified by QualBrowser software 1.4 (THERMO).

Two quantum chemistry program packages, Spartan'10 (Wavefunction, Inc.) and Q-Chem 3.2 [38], were used to calculate the energies of protonated isomer precursors. The temperature and pressure were set to 443.15 K, and 2.2 × 10^-8 ^atm, according to the instrument read-outs of the ion transfer tube temperature and the pressure in the ion trap. The calculations included the following principle steps. One hundred thousand conformers for each structure were examined, and the 1000 lowest energy conformers for each possible protonation site were kept, using the molecular mechanics MMFF force field and Monte Carlo Sampling. Next, semi-empirical RM1 and quantum mechanical DFT geometry optimizations and vibrational analyses were performed. Derived entropic and enthalpic corrections were included to convert the internal energy into free energies, which were used to calculate the Boltzmann distribution of the protonated isomers. The DFT calculations used the B3LYP functional and the 6-31G** basis set. The details of this computational algorithm constitute the results of the presented work and are given in the next section.

## Results

### Experimental data

**Figure 1:**
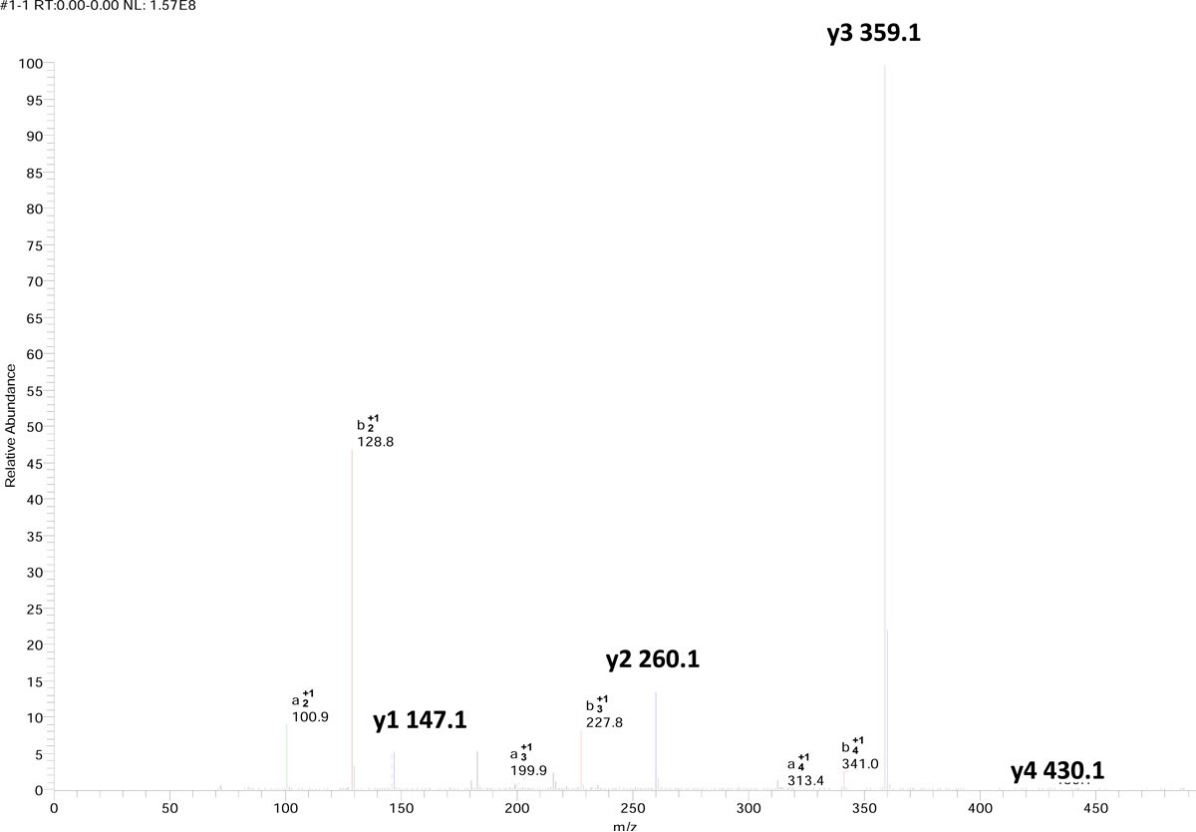
**GAVLK spectrum**. An example of an annotated MSMS spectrum of the GAVLK doubly charged peptide collected by an LCQ Deca XP Plus (THERMO) ion trap mass spectrometer, using the CID fragmentation method.

**Figure 2:**
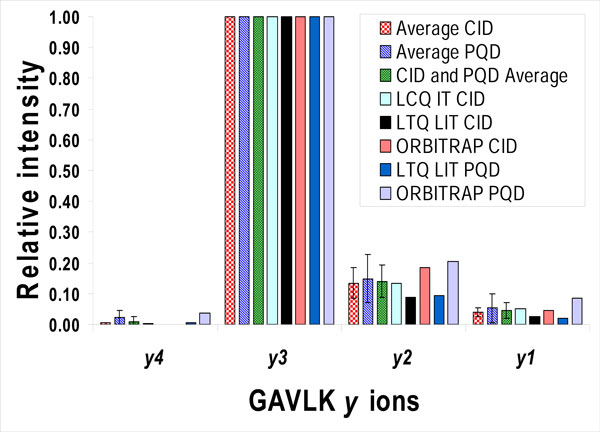
**Y-ions intensities**. Relative intensities of the *y *series ions of the GAVLK doubly charged peptide, as measured by LCQ Deca XP Plus and LTQ-Orbitrap Velos (THERMO) mass spectrometers. Peak intensities were quantified by QualBrowser 1.4 (THERMO), and the intensity of the *y*-3 ion was chosen to have a reference value of 1.0 for all measurements. **IT**-ion trap, **LIT-**linear ion trap, **CID**-Collision Induced Decay, **PQD**-Pulsed-Q Dissociation. The error bars represent the standard deviation (SD). Only the average values show SD (for average CID *y*4 ion, the SD is 0.0004, and as such the error bar is practically invisible; the reference *y*3 ions have SD = 0). The ion intensities from particular measurements were accumulated for five minutes and saved as one combination of *m/z *and relative intensity values (see additional file 1), so no SD is given.

The synthetic penta-peptide GAVLK was chosen as a model system due to its small number of amino acids and its overall low count of atoms. At the same time, it represents a realistic peptide that could be found in a tryptic digest of a protein sample. MSMS spectra of doubly charged GAVLK (Figure 1) were collected on electrospray ion trap mass spectrometers (see additional file 1 for all ions *m/z *and relative intensities values). Out of all the possible sequence informative ions, four *y *ions (1-4), three *b *ions (2-4), and three *a *ions (2-4) were detected. Regardless of the kind of mass detector or the fragmentation mode, the relative ion intensities were preserved in both order and ratios, from the most intense to the least intense ion pairs: *y3-b2*, *y2-b3, y1-b4*, and *y4-(b1 *not detected). As the most intense ion in all measurements, the *y3 *ion was chosen to be the reference ion and was assigned a relative intensity of 1.0. Due to their better stability [6] and consequent dominant spectral intensity, the *y *series ions were examined quantitatively in this study (Figure 2).

### The algorithm for the theoretical calculations

**Figure 3:**
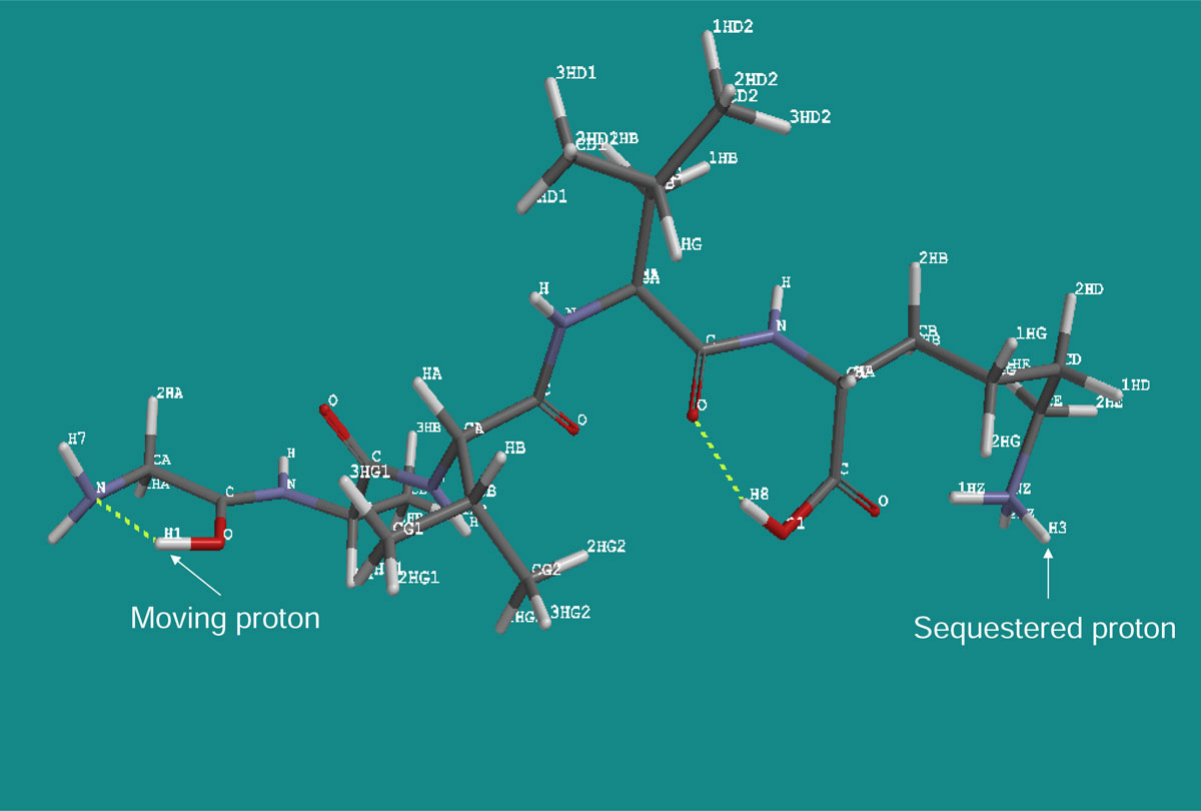
**GAVLK_C-OH_2 isomer**. An example of a GAVLK penta-peptide isomer designated as GAVLK_C-OH_2. It is protonated at the amide oxygen in position 2 and at the amine group of the C-terminal lysine. Arrows point to the moving and sequestered protons.

**Figure 4:**
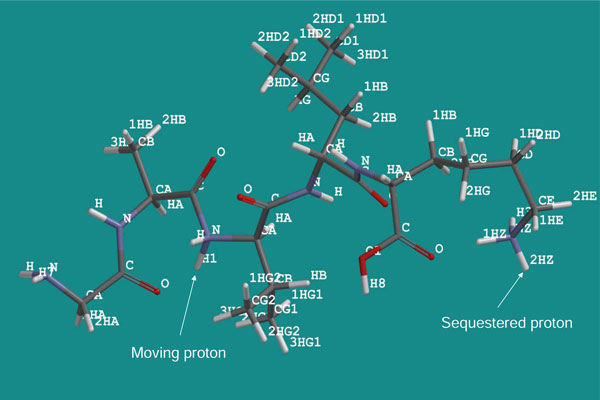
**GAVLK_NH_3 isomer**. An example of a GAVLK penta-peptide isomer designated as GAVLK_NH_3. It is protonated at the amide nitrogen in position 3 and at the amine group of the C-terminal lysine. Arrows point to the moving and sequestered protons.

To determine the fragment ions' intensities theoretically, we followed our hypothesis that, since the fragmentation is charge driven [6] by the protonation of the oxygen and/or nitrogen atoms in the backbone amide bond, the order of intensities of the fragment ions should depend on the thermodynamic stability of the particular protonated isomers of the parent ion present in the ion trap just prior to the fragmentation. The isomers were designated as GAVLK_C-OH_1 to GAVLK_C-OH_5, according to the position of the moving proton, starting with position one as the N-terminal amine group and advancing through the four amide bond oxygens (positions 2-5), in the direction from the N- to the C-terminus (Figure 3). Similarly, isomers protonated at amide nitrogens were labelled GAVLK_NH_1 to GAVLK_NH_5 (Figure 4). While one proton was allowed to move along the protonation sites of the peptide backbone, the second proton was sequestered on the amine group of the C-terminal lysine (K) residue in all calculations (see Discussion).

To calculate isomer energies and consequently their Boltzmann distribution, the following algorithm was developed. For the first steps we used the SPARTAN'10 (Wavefunction, Inc) software and the molecular mechanics MMFF force field to generate as many conformers as possible for each protonation site. As the peptides have rotatable bonds along the backbone and on each side chain, the number of possible conformers for even a short peptide chain is quite large. SPARTAN includes the ability to automatically generate and calculate these conformers. The approach the program uses is to randomly choose a bond to rotate and an amount to rotate it. From this starting point the geometry is optimized to find a local minimum structure close to the starting randomly-generated structure. This procedure is then repeated until either a predefined number of structures have been generated or until all possible structures have been examined. In the calculation 100,000 conformers were examined, and the 1,000 lowest energy conformers were kept. Next, an RM1 semi-empirical geometry optimization was performed on each of the conformers. RM1 was chosen over AM1 as the preferred semi-empirical method for this problem based on preliminary trials (see SM Table one in additional file 1) This calculation involves changing the geometry of the molecule to find a nearby structure that minimizes the energy. Low-energy RM1 structures (within 40 kJ.mol^-1 ^of the lowest energy structure) were used as starting geometries for DFT geometry optimizations. The target conformer energy window for the DFT results is 20 kJ.mol^-1^, but since the DFT calculation changes the conformers' energy values and their order greatly, a wider energy window as a starting point is necessary. The 20 kJ.mol^-1 ^threshold is justified by the realization that the Boltzmann distribution for two structures differing in energy by 20 kJ.mol^-1 ^results in approximately 0.6% abundance of the higher energy state at 443.15 K (the experimental condition). Prior to and after the DFT calculations, the conformers with energies within 0.03 kJ.mol^-1 ^of each other were reviewed for structure similarity by aligning the molecules (using a built-in function of SPARTAN). In the case of identical structures (Align Score > 0.98), only one structure was kept to avoid any conformer appearing more than once in the Boltzmann distribution.

The DFT calculations were divided into two steps - structure optimization and calculations of harmonic vibrational frequencies. The first step was performed by SPARTAN using the B3LYP functional and the 6-31G** basis set. The optimized geometries were used to create input files for vibrational analysis by Q-Chem 3.2 [38]. The vibrational frequency data were used to derive entropic and enthalpic corrections that converted the internal energy from the geometry optimization calculations into Gibbs free energies. The free energies were used to calculate the Boltzmann distribution of protonated isomer precursors, which should correspond with experimental relative ion intensities (see additional file 1 for algorithm schematic flowchart and work spread sheets).

### Theoretical calculations vs. Experimental data

**Figure 5:**
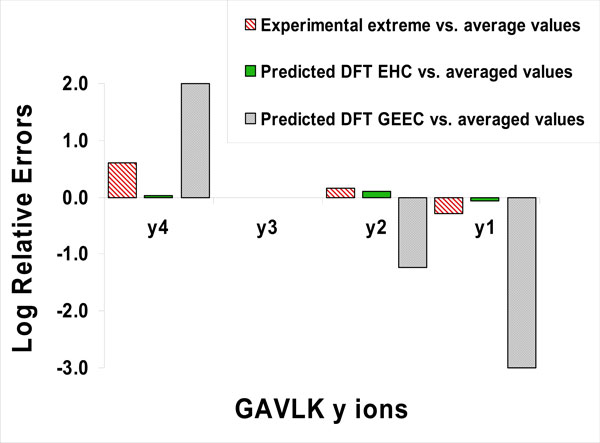
**Error plot**. The accuracy of predictions of relative intensities of *y *ions in MSMS spectra of the doubly charged GAVLK peptide. The best performing conformational space sampling method (**DFT EHC**; DFT energies with enthalpic corrections) and the worst performing method (**DFT GEEC**; DFT Gibbs free energy of single equilibrium conformer for each isomer) are presented in comparison to averaged experimental data. The Log Relative Errors were calculated as log_10 _(predicted relative intensity/measured relative intensity) for each ion. To demonstrate that our best predictions were within the experimental variation of the measurements, the differences between the most extreme experimental values and the averaged values are also presented. The intensity of the *y*3 ion was predicted as the highest according to the experiment, in which the *y*3 was chosen to have a reference value of 1.0, and consequently its logErr values are zero.

Ion intensities based on Boltzmann distributions derived from the amide oxygen isomers provided initial results that were off by 5-7 orders of magnitude compared to experimental ion intensities (see SM Table one in additional file 1). However, when amide nitrogen protonated isomers were considered, the DFT energy calculations including the low energy conformers for each ion provided predictions of y-ions relative intensities in agreement with the experiment. As shown in Table 1 and Figure 5, we were able to a) predict the *y *and *b *ions' intensity *order *in concert with the experimental values, and b) predict *relative intensities *of *y *ions with errors not exceeding experimental variation.

**Table 1:** Experimental and predicted ion intensities in the MSMS spectra of the doubly charged GAVLK peptide.

Protonated isomers	ions	Experimental intensity order	Predicted order*	Averaged experimental relative intensity CID and PQD	Predicted relative intensity DFT EHC	Predicted relative intensity DFT E	Predicted relative intensity DFT GE	Predicted relative intensity DFT GEEC
**GAVLK_NH_2**	***y4***	4	4	**0.0095**	**0.0102**	0.0203	0.0186	1.0500(1)
**GAVLK_NH_3**	***y3***	1	1	**1.0000**	**1.0000**	1.0000	1.0000	1.0000(2)
**GAVLK_NH_4**	***y2***	2	2	**0.1405**	**0.1780**	0.2902	0.0945	0.0083(3)
**GAVLK_NH_5**	***y1***	3	3	**0.0451**	**0.0382**	0.0438	0.0003	0.00004(4)

**GAVLK_NH_2**	***b1***	ND	4					
**GAVLK_NH_3**	***b2***	1	1	**Sum of logErr:**	**0.2076**	0.6549	2.6428	**6.3255**
				
**GAVLK_NH_4**	***b3***	2	2					
**GAVLK_NH_5**	***b4***	3	3					

Boltzmann distributions were based on the free energies of all the low energy (within 20 kJ.mol^-1 ^of the lowest) conformers calculated as follows: **EHC - **DFT energy with enthalpic corrections; **E **- DFT energy; **GE**- Gibbs free energy; except for **GEEC**, when the Gibbs free energy of a single equilibrium conformer for each isomer was used. The calculations for GAVLK_NH_1 isomers energies were not considered, because that particular protonation does not lead to fragmentation and as such is inconsequential. **ND **- not detected; **logErr **- decadic logarithm of relative error.

* The predicted order for the **GEEC **calculations is given in parenthesis in the pertinent column.

## Discussion

Initially, the same principal calculations (MMFF-RM1-DFT-Boltzmann distribution) were performed for one equilibrium conformer for each protonated isomer. But as shown above, that proved to be not sufficient. A significant improvement in the agreement with experiment occurs when the conformational space for each isomer was examined via the sum of DFT conformer weights. Clearly, careful conformational sampling and summation matters (notice the logarithmic scale in Figure 5 and the Table 1 logErr parameter, necessary to depict and stress the several orders of magnitude differences in the accuracies of prediction). The peptide needs to be presented to the computational algorithm as an amino acid sequence, then a population of protonated isomers, and finally as a large group of conformers for each isomer.

For all calculations we adhered to a unifying presumption - one of the protons moved along the protonation sites of the peptide backbone, while the other proton was sequestered on the C-terminal lysine (K) residue. This is a well-accepted consensus [3], and for example Bythell *et al. *[36] found that *b*-2 ions favoured oxazolone structures, suggesting that the doubly charged peptides undergo fragmentation via a *b*-*y *fragmentation pathway, contrary to the cyclic peptide pathway favoured by singly charged peptides [39]. Our results are in concert with this finding, and they validate the starting point for the calculations - the population of linear doubly protonated peptide isomers.

The surprising outcome of this study is that the amide oxygen protonated isomers did not yield accurate predictions, whereas the protonated amide nitrogen populations gave very good agreement with measured intensities. Despite the amide oxygen protonation being energetically favoured [27,36], the protonation of an amide nitrogen ultimately leads to fragmentation, due to weakening the amide bond and to making the carbon centre of the amide bond more positive and therefore a better target for nucleophilic attack [27]. As such, amide nitrogen protonated isomers appear to play the decisive role in determining the fragmentation products, and this is supported by our results.

The most accurate prediction of ion intensities was obtained by utilizing DFT energies with enthalpic corrections but not entropic corrections included. Even the DFT energies by themselves provided results in good agreement with the experimental results. However, the free energies (including also the entropic corrections) led to less accurate predictions, especially for the intensity of the *y-1 *ion. The likely reason for this inaccuracy can be found in the harmonic approximation that is used in standard quantum chemical calculations to determine the vibrational frequencies and thus the vibrational enthalpic and entropic corrections. In general, the harmonic approximation is most suspect for the very low frequency modes, which are present in large numbers in floppy molecules, such as the ones studied here. The vibrational entropy correction is dominated by these same low frequency modes, and therefore it is not unusual for entropic corrections for large, floppy molecules to be inaccurate. On the other hand, vibrational enthalpic corrections are dominated by the high frequency modes and so do not suffer from the same problems. Ignoring entropic corrections seem to be an easy fix, since the results with enthalpic corrections are accurate enough, and the entropies of isomers such as these tend to be quite similar in practice.

Improving upon the accuracy of these predictions, including studying potentially better entropy predictions, will be the subject of further studies. In addition, we will investigate use of other DFT functionals, such asM06-2X and ωB97X-D. The selection of the B3LYP functional in this study was based on its combined performance in geometries and thermochemistry calculations [40]. Compared to using different functionals, though, using larger basis sets promises much smaller accuracy gains, while greatly increasing the time needed for the calculations.

We realize that our algorithm and hypothesis need to be validated on more than one peptide. This study is a "proof of concept" and is limited to just the doubly charged GAVLK peptide. With that, a concern may arise that the theoretical prediction might agree with the experimental observation by chance alone. The odds of randomly picking the correct intensity order for the four *y *ions are 1 in 24, or 4.2%. However, the likelihood of a random match gets progressively lower when predicting the relative ion intensities. The largest discrepancy between the theoretical prediction and the average experimental relative intensity was less than 4%. So in the worst case, the chance that our results arise from a random match is approximately 1:25 × 1:25 × 1:25 (for three intensity values relative to the most intense ion), or about 1 in 15,625. Still, it remains to be shown if the approach taken here will work for other peptides, especially those containing amino acids that have a great effect on fragmentation, such as proline and glycine [41]. In the case of glycine, both our experimental and theoretical results are in agreement with previous observations (extremely weak cleavage C-terminal to glycine [41]); nevertheless we intend to explore other permutations of the GAVLK sequence, longer peptides, and sequences including proline.

The presented and planned calculations are, however, extremely costly. Currently, it takes approximately 100 processor hours to complete the calculations for one conformer (hundreds of "DFT" conformers were examined in this study), and so the calculations necessary for this manuscript required tens of thousands of CPU hours. On the other hand, despite the considerable effort necessary to generate these preliminary results, we do find them to be unique and promising.

## Conclusions

Using tools of computational chemistry, we were able to predict ion intensities in the MSMS spectra of the doubly charged GAVLK penta-peptide. The results suggest that the peptide fragmentation process in the ion trap mass spectrometer is predominantly driven by the thermodynamic stability of the N-protonated precursor ions formed upon ionization of the sample. A combination of precursor conformational sampling, along with accurate energy calculations of the conformers and their Boltzmann distribution, is necessary to achieve agreement between experiment and prediction. In addition to the intrinsic intellectual value, the presented findings are worthy of further pursuit, because they represent first steps on the so far unexplored avenue of integrating statistical approaches with fundamental quantum mechanical calculations for more confident molecular identification. Currently, such computational approach is too time-consuming to be performed on thousands of potential peptide matches for spectra in high through-put experiments. However, it is reasonable to expect that exponential growth in computing power will continue and quantum calculations will potentially become practical, at least for targeted molecular analysis.

## List of abbreviations used

MSMS: tandem mass spectrometry; *m/z*: mass to charge ratio; DFT: density functional theory; ESI: electrospray ionization; aa: amino acids; IT: ion trap; LIT: linear ion trap; CID: Collision Induced Decay; PQD: Pulsed-Q Dissociation; EHC: DFT energy with enthalpic corrections; E: DFT energy; GE: Gibbs Free Energy; GEEC: Gibbs Free Energy of a single equilibrium conformer; ND: not detected; logErr: decadic logarithm of relative error.

## Competing interests

The authors declare that they have no competing interests.

## Authors' contributions

TP conceived of study, collected and analyzed mass spectrometry data, performed the computations, and was the primary manuscript writer. SRG designed the original computational algorithm used and helped interpret the results. Both authors read and approved the final manuscript.

## Supplementary Material

Additional file 1 **Supplementary materials**. Click here for file
