# Supplementary material for: Calculations of relative intensities of fragment ions in the MSMS spectra of a doubly charged penta-peptide
Source: BMC Bioinformatics. 2012 Sep 11;13(Suppl 15):S13. doi: 10.1186/1471-2105-13-S15-S13 (PMC3439735; doi:10.1186/1471-2105-13-S15-S13)
Supplement: Additional file 1 — Supplementary materials. [file 1471-2105-13-S15-S13-S1.pdf]

Pechan and Gwaltney, 2012, Supplemental Materials.

1. Ion lists in MSMS spectra of doubly charged GAVLK peptide (partial "top intensity ions" lists are shown, selected with a threshold to include all y ions).

LCQ Ion Trap CID (MS analyzer and mode of fragmentation)
GAVLK_average.RAW (file name)
+ c NSI Full ms2 244.30@cid35.00 [65.00-500.00]

m/z	Intensity	Relative
72.04	939403.0	0.60
100.90	13986980.0	8.90
128.81	73461264.0	46.74
129.89	4837486.0	3.08
147.00	7856074.0	5.00
180.17	1763917.0	1.12
182.86	8111029.0	5.16
199.11	1097774.0	0.70
215.68	3513231.0	2.24
216.71	1645193.0	1.05
227.83	12618236.0	8.03
228.91	827352.0	0.53
234.95	852616.0	0.54
260.06	20861544.0	13.27
261.12	2203989.0	1.40
312.88	1816183.0	1.16
341.04	3753277.0	2.39
359.10	157164352.0	100.00
360.13	34382288.0	21.88
361.16	1155301.0	0.74

LTQ IT CID (MS analyzer and mode of fragmentation)
IT_IT_CID_110310190542.raw (file name)
ITMS + c NSI Full ms2 244.30@cid35.00 [65.00-1000.00]

m/z	Intensity	Relative
72.21	1672.9	0.12
84.23	767.6	0.06
101.16	139606.5	10.06
102.17	8210.2	0.59
103.21	452.7	0.03
129.11	466152.0	33.58
130.09	28906.3	2.08
131.14	2588.0	0.19
147.14	34816.4	2.51
148.19	2531.9	0.18
149.10	763.5	0.06
180.26	16733.8	1.21

181.06	921.5	0.07
183.16	15689.0	1.13
184.20	2126.6	0.15
185.22	246.2	0.02
186.16	402.8	0.03
197.24	1742.2	0.13
198.27	372.3	0.03
200.16	4082.3	0.29
201.21	559.6	0.04
215.82	16914.1	1.22
216.61	1201.7	0.09
221.82	2486.4	0.18
222.54	267.7	0.02
225.29	862.7	0.06
226.29	2849.0	0.21
227.38	620.3	0.04
228.18	88925.2	6.41
229.20	10255.4	0.74
230.24	1074.7	0.08
235.35	8909.4	0.64
236.14	727.9	0.05
260.29	120598.2	8.69
261.30	18024.0	1.30
262.34	1895.4	0.14
296.37	1380.8	0.10
297.37	250.7	0.02
313.33	6950.1	0.50
314.37	1226.7	0.09
341.33	27303.9	1.97
342.35	4503.7	0.32
343.38	666.3	0.05
359.50	1388026.5	100.00
360.36	178592.3	12.87
361.36	25159.2	1.81
362.40	2240.9	0.16
430.44	1548.6	0.11
431.44	450.5	0.03
440.43	319.1	0.02

ORBITRAP FTMS CID (MS analyzer and mode of fragmentation)
FTMS_CID_average.raw (file name)
FTMS + c NSI Full ms2 244.16@cid35.00 [65.00-1000.00]

m/z	Intensity	Relative
66.13	458.3	0.06
72.08	3026.8	0.41
100.78	658.9	0.09
101.07	648.2	0.09
101.07	1951.9	0.26
101.07	939.7	0.13

101.07	601.7	0.08
101.07	222361.9	29.81
101.07	5119.0	0.69
101.07	1501.5	0.20
101.07	2067.9	0.28
101.07	869.9	0.12
101.37	677.3	0.09
102.07	4207.7	0.56
106.13	477.7	0.06
128.64	798.4	0.11
128.69	402.4	0.05
129.06	674.9	0.09
129.06	368.5	0.05
129.06	1738.3	0.23
129.06	1467.1	0.20
129.07	260966.0	34.98
129.07	6163.4	0.83
129.07	1773.1	0.24
129.07	2092.0	0.28
129.07	440.1	0.06
129.07	388.1	0.05
129.07	949.6	0.13
129.10	6129.5	0.82
129.44	413.8	0.06
129.49	808.9	0.11
130.07	7723.0	1.04
130.09	5506.8	0.74
136.40	358.1	0.05
147.11	33389.1	4.48
147.11	785.5	0.11
148.12	396.8	0.05
155.12	2481.5	0.33
171.11	771.9	0.10
180.14	28173.0	3.78
180.14	668.1	0.09
180.64	2138.5	0.29
183.11	35884.0	4.81
183.11	845.8	0.11
184.11	920.6	0.12
197.16	459.4	0.06
200.14	3402.8	0.46
215.65	36653.2	4.91
216.16	3622.8	0.49
221.65	2266.3	0.30
228.13	63246.1	8.48
229.14	2015.3	0.27
235.16	1019.5	0.14
242.18	449.9	0.06
258.98	398.4	0.05
260.19	882.1	0.12
260.19	456.0	0.06

260.20	137622.	18.45
260.20	1122.2	0.15
260.20	423.0	0.06
261.20	8829.2	1.18
261.42	394.9	0.05
296.19	621.9	0.08
313.22	3702.5	0.50
341.22	16737.5	2.24
341.25	423.9	0.06
342.22	886.8	0.12
357.29	2399.0	0.32
357.52	1303.9	0.17
359.21	563.0	0.08
359.21	358.8	0.05
359.22	408.0	0.05
359.22	496.5	0.07
359.22	452.4	0.06
359.23	760.0	0.10
359.23	974.9	0.13
359.24	1203.5	0.16
359.24	2151.4	0.29
359.25	1606.3	0.22
359.25	4485.2	0.60
359.26	4836.9	0.65
359.26	745956.9	100.00
359.27	1919.3	0.26
359.27	5093.2	0.68
359.28	1717.6	0.23
359.28	1784.4	0.24
359.29	871.6	0.12
359.29	929.4	0.12
359.30	925.8	0.12
359.30	659.6	0.09
359.31	538.7	0.07
360.25	547.3	0.07
360.26	580.9	0.08
360.27	103139.2	13.83
360.27	505.1	0.07
360.28	547.9	0.07
361.02	1340.4	0.18
361.25	2441.1	0.33
394.33	558.8	0.07
430.30	728.4	0.10

LTQ IT PQD (MS analyzer and mode of fragmentation)
IT_IT_PQD_110310190542.raw (file name)
ITMS + c NSI Full ms2 244.30@pqd35.00 [65.00-1000.00]

m/z	Intensity	Relative
101.21	10384.7	0.77

129.15	106977.8	7.96
130.17	6150.1	0.46
147.19	27194.2	2.02
180.23	8700.9	0.65
183.19	16087.8	1.20
215.77	37221.7	2.77
228.22	47044.0	3.50
235.40	6372.5	0.47
242.34	17141.7	1.28
243.49	7877.9	0.59
244.50	1337766.5	99.51
245.12	628921.3	46.78
260.29	127446.1	9.48
261.30	7962.2	0.59
341.34	10530.4	0.78
359.57	1344342.3	100.00
360.37	69993.4	5.21
361.37	14556.1	1.08
430.39	7482.3	0.56

ORBITRAP FTMS PQD ((MS analyzer and mode of fragmentation))
FTMS_PQD_110310190542.raw (file name)
FTMS + c NSI Full ms2 244.16@pqd35.00 [65.00-1000.00]

m/z	Intensity	Relative
129.10	9745.2	3.35
130.09	1997.7	0.69
147.11	24807.4	8.54
185.16	5301.3	1.82
213.16	6447.8	2.22
225.16	956.3	0.33
228.13	1624.7	0.56
242.18	7856.3	2.70
243.17	1730.4	0.60
260.19	59091.2	20.34
261.20	5468.2	1.88
324.23	1191.2	0.41
359.25	1612.1	0.55
359.26	290531.6	100.00
359.27	1456.7	0.50
359.27	1741.1	0.60
360.27	59273.3	20.40
361.25	940.0	0.32
430.30	10987.3	3.78
431.30	1338.8	0.46

SM Table 1. Relative Boltzmann distributions for *y* series ions of GAVLK doubly charged paptide, as calculated by AM1, RM1, and DFT methods vs. experimentally observed values.

**Molecule**	ions	**Relative ion intensities Experimental CID and PQD AVERAGED (Order)**	Relative BOLTZMANN Distribution based on **AM1 G (kJ/mol)** for low conformers **within 20kJ/mol range** 443.15 K 2.2e-8 ATM	Relative BOLTZMANN Distribution based on **RM1 G (kJ/mol)** for low conformers **within 20kJ/mol range** 443.15 K 2.2e-8 ATM	Relative BOLTZMANN Distribution based on **DFT G (kJ/mol)** for equilibrium conformer 443.15 K 2.2e-8 ATM
**GALVK_NH_2**	y4	**0.0095 (4.)**	0.0499 (3.)	0.0909 (3.)	1.0500 (1.)
**GAVLK_NH_3**	y3	**1.0000 (1.)**	1.0000 (1.)	1.0000 (1.)	1.0000 (2.)
**GAVLK_NH_4**	y2	**0.1405 (2.)**	0.3760 (2.)	0.0714 (2.)	0.0083 (3.)
**GAVLK_NH_5**	y1	**0.0451 (3.)**	0.00001 (4.)	0.0010 (4.)	0.00004 (4.)
**GAVLK_C-OH_2**	y4	**0.0095 (4.)**	53.742 (1.)	49.921 (1.)	154.6405 (1.)*
**GAVLK_C-OH_3**	y3	**1.0000 (1.)**	1.0000 (2.)	1.0000 (2.)	1.0000 (2.)*
**GAVLK_C-OH_4**	y2	**0.1405 (2.)**	0.0054 (3.)	0.1099 (3.)	0.000048 (3.)*
**GAVLK_C-OH_5**	y1	**0.0451 (3.)**	0.0002 (4.)	0.0070 (4.)	0.0000000059 (4.)*

* DFT energy only

Calculation Flowchart

Molecular mechanics MMFF force field and Monte Carlo conformers sampling (max 100.000 to examine, maximum 1,000 kept)

↓

RM1 semi-empirical geometry optimization on each of kept conformers

↓

Low-energy RM1 conformers (within 40 kJ/mol) kept

↓

Duplicate conformers with equal energies (within 0.03 kJ/mol) and identical structures (Align Score >0.98) removed

↓

The **DFT** geometry optimization using the **6-31G**** basis set and **B3LYP** functionals

↓

Calculations of harmonic vibrational frequencies, using **DFT 6-31G**** basis set and **B3LYP** functionals

↓

Entropic and enthalpic corrections derived from vibrational frequency data to convert the internal energy into **free energies**

↓

Duplicate conformers with equal energies (within 0.03 kJ/mol) and identical structures (Align Score >0.98) removed

↓

Free energies were used to calculate the **Boltzmann distribution** of protonated isomer precursors

Spreadsheet for DFT Energy lowest conformers with Enthalpic Corrections (DFT EHC)

Molecule	Conformers	DFT Energy kJ/mol	DFT EHC kJ/mol 443.15 K 2.2e-8 ATM	Boltzman Distribution	ions	Relative ion intensities CID PQD Average	Relative ion intensities Predicted
GAVLK_NH_2	Molecule003	-4317313.02	-4315285.72860	0.00038			
GAVLK_NH_2	Molecule024	-4317324.04	-4315299.00910	0.01400	**y4**	**0.00950**	**0.0102**
GAVLK_NH_2	Molecule041	-4317312.79	-4315289.59170	0.00109	**y3**	**1.00000**	**1.0000**
GAVLK_NH_2	Molecule050	-4317310.68	-4315287.41475	0.00060	**y2**	**0.14050**	**0.1780**
GAVLK_NH_2	Molecule121	-4317311.43	-4315288.39906	0.00079	**y1**	**0.04510**	**0.0382**
GAVLK_NH_2	Molecule202	-4317319.32	-4315294.27237	0.00387			
							**SUM BOLTZ**
GAVLK_NH_3	Molecule023	-4317332.02	-4315307.66810	0.14682	**y4**		**0.0207**
GAVLK_NH_3	Molecule050	-4317323.60	-4315304.99573	0.07109	**y3**		**2.0246**
GAVLK_NH_3	Molecule051	-4317328.27	-4315309.32264	0.23004	**y2**		**0.3604**
GAVLK_NH_3	Molecule068	-4317321.34	-4315296.95344	0.00801	**y1**		**0.0773**
GAVLK_NH_3	Molecule069	-4317324.54	-4315301.28730	0.02598			
GAVLK_NH_3	Molecule072	-4317321.22	-4315297.03846	0.00820			
GAVLK_NH_3	Molecule079	-4317321.28	-4315296.97712	0.00807			
GAVLK_NH_3	Molecule122	-4317337.45	-4315314.73704	1.00000			
GAVLK_NH_3	Molecule146	-4317321.16	-4315297.08306	0.00830			
GAVLK_NH_3	Molecule199	-4317327.64	-4315309.16125	0.22019			
GAVLK_NH_3	Molecule235	-4317317.86	-4315293.72866	0.00334			
GAVLK_NH_3	Molecule260	-4317321.25	-4315300.31106	0.01994			
GAVLK_NH_3	Molecule268	-4317321.11	-4315297.08326	0.00830			
GAVLK_NH_3	Molecule281	-4317330.25	-4315305.48570	0.08120			
GAVLK_NH_3	Molecule288	-4317327.36	-4315307.68881	0.14765			
GAVLK_NH_3	Molecule311	-4317317.93	-4315298.66466	0.01275			
GAVLK_NH_3	Molecule324	-4317321.31	-4315296.96528	0.00804			
GAVLK_NH_3	Molecule329	-4317319.69	-4315299.65898	0.01670			
GAVLK_NH_4	Molecule001	-4317326.56	-4315299.64212	0.01663			
GAVLK_NH_4	Molecule002	-4317323.51	-4315295.67582	0.00567			
GAVLK_NH_4	Molecule034	-4317316.36	-4315291.38166	0.00177			
GAVLK_NH_4	Molecule035	-4317316.26	-4315292.93235	0.00269			
GAVLK_NH_4	Molecule068	-4317326.96	-4315300.60610	0.02160			
GAVLK_NH_4	Molecule073	-4317317.69	-4315294.16970	0.00376			
GAVLK_NH_4	Molecule113	-4317323.48	-4315295.54959	0.00548			
GAVLK_NH_4	Molecule184	-4317327.86	-4315301.30520	0.02611			
GAVLK_NH_4	Molecule185	-4317326.72	-4315304.38360	0.06021			
GAVLK_NH_4	Molecule187	-4317317.54	-4315292.55894	0.00243			
GAVLK_NH_4	Molecule188	-4317322.39	-4315303.75644	0.05078			
GAVLK_NH_4	Molecule194	-4317314.83	-4315291.54824	0.00185			
GAVLK_NH_4	Molecule195	-4317325.79	-4315299.26960	0.01503			
GAVLK_NH_4	Molecule196	-4317319.03	-4315296.12458	0.00640			
GAVLK_NH_4	Molecule206	-4317326.66	-4315299.64170	0.01662			
GAVLK_NH_4	Molecule208	-4317314.79	-4315291.48291	0.00182			
GAVLK_NH_4	Molecule209	-4317319.09	-4315292.20141	0.00221			
GAVLK_NH_4	Molecule217	-4317322.34	-4315294.13763	0.00373			
GAVLK_NH_4	Molecule218	-4317315.09	-4315287.94529	0.00070			

GAVLK_NH_4	Molecule222	-4317316.31	-4315293.05310	0.00278
GAVLK_NH_4	Molecule225	-4317326.54	-4315299.60957	0.01648
GAVLK_NH_4	Molecule227	-4317318.90	-4315296.02805	0.00623
GAVLK_NH_4	Molecule229	-4317323.29	-4315300.09588	0.01880
GAVLK_NH_4	Molecule231	-4317316.63	-4315293.93508	0.00353
GAVLK_NH_4	Molecule234	-4317318.19	-4315295.41010	0.00527
GAVLK_NH_4	Molecule237	-4317326.67	-4315299.34627	0.01534
GAVLK_NH_4	Molecule239	-4317327.87	-4315301.37440	0.02660
GAVLK_NH_4	Molecule253	-4317325.29	-4315300.30893	0.01992
GAVLK_NH_5	Molecule001	-4317307.74	-4315281.75515	0.00013
GAVLK_NH_5	Molecule013	-4317325.49	-4315302.40885	0.03523
GAVLK_NH_5	Molecule027	-4317312.85	-4315286.26684	0.00044
GAVLK_NH_5	Molecule081	-4317307.57	-4315281.54750	0.00012
GAVLK_NH_5	Molecule092	-4317319.10	-4315298.07319	0.01086
GAVLK_NH_5	Molecule118	-4317324.94	-4315297.29410	0.00879
GAVLK_NH_5	Molecule131	-4317315.86	-4315300.61804	0.02167
GAVLK_NH_5	Molecule183	-4317305.25	-4315278.21915	0.00005

Spreadsheet for DFT Energy lowest conformers (DFT E)

Molecule	Conformers	DFT Energy kJ/mol	Boltzman Distribution	ions	Relative ion intensities CID PQD Average	Relative ion intensities Predicted
GAVLK_NH_2	Molecule003	-4317313.02	0.00126			
GAVLK_NH_2	Molecule024	-4317324.04	0.02515	**y4**	**0.00950**	**0.0203**
GAVLK_NH_2	Molecule041	-4317312.79	0.00119	**y3**	**1.00000**	**1.0000**
GAVLK_NH_2	Molecule050	-4317310.68	0.00067	**y2**	**0.14050**	**0.2902**
GAVLK_NH_2	Molecule121	-4317311.43	0.00082	**y1**	**0.04510**	**0.0438**
GAVLK_NH_2	Molecule202	-4317319.32	0.00699			
						**SUM BOLTZ**
GAVLK_NH_3	Molecule023	-4317332.02	0.21934	**y4**		0.0361
GAVLK_NH_3	Molecule027	-4317321.19	0.01160	**y3**		1.7801
GAVLK_NH_3	Molecule050	-4317323.60	0.02232	**y2**		0.5165
GAVLK_NH_3	Molecule051	-4317328.27	0.07927	**y1**		0.0779
GAVLK_NH_3	Molecule068	-4317321.34	0.01209			
GAVLK_NH_3	Molecule069	-4317324.54	0.02880			
GAVLK_NH_3	Molecule072	-4317321.22	0.01170			
GAVLK_NH_3	Molecule079	-4317321.28	0.01189			
GAVLK_NH_3	Molecule122	-4317337.61	1.00000			
GAVLK_NH_3	Molecule146	-4317321.16	0.01151			
GAVLK_NH_3	Molecule176	-4317327.60	0.06609			
GAVLK_NH_3	Molecule199	-4317327.64	0.06681			
GAVLK_NH_3	Molecule260	-4317321.25	0.01179			
GAVLK_NH_3	Molecule268	-4317321.11	0.01135			
GAVLK_NH_3	Molecule281	-4317329.93	0.12438			
GAVLK_NH_3	Molecule288	-4317327.36	0.06192			
GAVLK_NH_3	Molecule294	-4317317.88	0.00473			
GAVLK_NH_3	Molecule311	-4317317.93	0.00479			
GAVLK_NH_3	Molecule324	-4317321.31	0.01199			
GAVLK_NH_3	Molecule329	-4317319.69	0.00772			
GAVLK_NH_4	Molecule001	-4317326.56	0.04984			
GAVLK_NH_4	Molecule002	-4317323.51	0.02178			
GAVLK_NH_4	Molecule034	-4317316.36	0.00313			
GAVLK_NH_4	Molecule035	-4317316.26	0.00304			
GAVLK_NH_4	Molecule068	-4317326.75	0.05247			
GAVLK_NH_4	Molecule073	-4317317.69	0.00449			
GAVLK_NH_4	Molecule113	-4317323.48	0.02160			
GAVLK_NH_4	Molecule184	-4317327.61	0.06627			
GAVLK_NH_4	Molecule187	-4317317.54	0.00431			
GAVLK_NH_4	Molecule188	-4317322.39	0.01607			
GAVLK_NH_4	Molecule194	-4317314.83	0.00207			
GAVLK_NH_4	Molecule195	-4317325.79	0.04044			
GAVLK_NH_4	Molecule196	-4317319.03	0.00646			
GAVLK_NH_4	Molecule204	-4317317.65	0.00444			
GAVLK_NH_4	Molecule208	-4317314.79	0.00204			
GAVLK_NH_4	Molecule209	-4317319.09	0.00656			
GAVLK_NH_4	Molecule217	-4317322.34	0.01585			

GAVLK_NH_4	Molecule218	-4317315.09	0.00222
GAVLK_NH_4	Molecule222	-4317316.31	0.00309
GAVLK_NH_4	Molecule227	-4317318.90	0.00623
GAVLK_NH_4	Molecule229	-4317323.29	0.02052
GAVLK_NH_4	Molecule231	-4317316.63	0.00337
GAVLK_NH_4	Molecule234	-4317318.19	0.00514
GAVLK_NH_4	Molecule237	-4317326.67	0.05135
GAVLK_NH_4	Molecule239	-4317327.73	0.06846
GAVLK_NH_4	Molecule253	-4317325.29	0.03531
GAVLK_NH_5	Molecule001	-4317307.74	0.00030
GAVLK_NH_5	Molecule013	-4317325.49	0.03728
GAVLK_NH_5	Molecule027	-4317312.85	0.00121
GAVLK_NH_5	Molecule081	-4317307.57	0.00029
GAVLK_NH_5	Molecule092	-4317319.10	0.00658
GAVLK_NH_5	Molecule118	-4317324.94	0.03211
GAVLK_NH_5	Molecule183	-4317305.25	0.00015

Spreadsheet for DFT Gibbs Energy lowest conformers (DFT GE)

Molecule	Conformers	DFT Energy kJ/mol	DFT Gibbs E kJ/mol 443.15 K 2.2e-8 ATM	Boltzman Distribution	ions	**Relative ion intensities CID PQD Average**	**Relative ion intensities Predicted**
GAVLK_NH_2	Molecule003	-4317313.10	-4315933.80350	0.0009			
GAVLK_NH_2	Molecule024	-4317324.04	-4315950.24597	0.0769	**y4**	**0.00950**	**0.0186**
GAVLK_NH_2	Molecule041	-4317312.79	-4315928.93797	0.0002	**y3**	**1.00000**	**1.0000**
GAVLK_NH_2	Molecule050	-4317310.68	-4315927.13741	0.0001	**y2**	**0.14050**	**0.0945**
					**y1**	**0.04510**	**0.0003**
GAVLK_NH_3	Molecule023	-4317331.84	-4315951.93730	0.1217			**SUM BOLTZ**
GAVLK_NH_3	Molecule051	-4317328.27	-4315946.59598	0.0285	**y4**		**0.0781**
GAVLK_NH_3	Molecule068	-4317321.34	-4315955.37510	0.3093	**y3**		**4.1906**
GAVLK_NH_3	Molecule069	-4317324.54	-4315956.04704	0.3711	**y2**		**0.3960**
GAVLK_NH_3	Molecule072	-4317321.22	-4315959.10535	0.8512	**y1**		**0.0013**
GAVLK_NH_3	Molecule079	-4317321.28	-4315956.77270	0.4519			
GAVLK_NH_3	Molecule122	-4317337.45	-4315955.30390	0.3034			
GAVLK_NH_3	Molecule235	-4317317.86	-4315949.14476	0.0570			
GAVLK_NH_3	Molecule260	-4317321.25	-4315942.60355	0.0097			
GAVLK_NH_3	Molecule268	-4317321.11	-4315959.69899	1.0000			
GAVLK_NH_3	Molecule281	-4317330.25	-4315949.55070	0.0637			
GAVLK_NH_3	Molecule288	-4317327.36	-4315946.28971	0.0263			
GAVLK_NH_3	Molecule311	-4317317.93	-4315953.92315	0.2085			
GAVLK_NH_3	Molecule324	-4317321.31	-4315956.21389	0.3883			
GAVLK_NH_4	Molecule001	-4317326.56	-4315947.41360	0.0356			
GAVLK_NH_4	Molecule002	-4317323.51	-4315942.91146	0.0105			
GAVLK_NH_4	Molecule068	-4317326.96	-4315949.46990	0.0623			
GAVLK_NH_4	Molecule113	-4317323.48	-4315941.43356	0.0070			
GAVLK_NH_4	Molecule184	-4317327.86	-4315946.65240	0.0290			
GAVLK_NH_4	Molecule185	-4317326.72	-4315938.28055	0.0030			
GAVLK_NH_4	Molecule195	-4317325.79	-4315951.83959	0.1185			
GAVLK_NH_4	Molecule206	-4317326.66	-4315948.29390	0.0453			
GAVLK_NH_4	Molecule208	-4317314.79	-4315933.47875	0.0008			
GAVLK_NH_4	Molecule209	-4317319.09	-4315942.46114	0.0093			
GAVLK_NH_4	Molecule217	-4317322.34	-4315941.32877	0.0068			
GAVLK_NH_4	Molecule225	-4317326.54	-4315947.19934	0.0336			
GAVLK_NH_4	Molecule237	-4317326.67	-4315942.93852	0.0106			
GAVLK_NH_4	Molecule239	-4317327.87	-4315945.81930	0.0231			
GAVLK_NH_4	Molecule253	-4317325.29	-4315931.92044	0.0005			
GAVLK_NH_5	Molecule001	-4317307.74	-4315916.28251	0.00001			
GAVLK_NH_5	Molecule003	-4317339.31	-4315930.68133	0.00038			
GAVLK_NH_5	Molecule013	-4317325.10	-4315922.13530	0.00004			
GAVLK_NH_5	Molecule027	-4317312.85	-4315923.35106	0.00005			
GAVLK_NH_5	Molecule066	-4317338.58	-4315927.89369	0.00018			
GAVLK_NH_5	Molecule081	-4317307.57	-4315918.40922	0.00001			
GAVLK_NH_5	Molecule092	-4317319.10	-4315932.14629	0.00057			
GAVLK_NH_5	Molecule118	-4317324.88	-4315920.69730	0.00003			

GAVLK_NH_5	Molecule131	-4317315.86	-4315920.59411	0.00002

Spreadsheet for DFT G Energy for single equilibrium conformer (DFT GEEC)

Molecule	DFT Energy kJ/mol	DFT Gibbs Energy kJ/mol 443.15 K 2.2e-8 ATM	**ions**	**Relative ion intensities CID PQD Average**	**Relative ion intensities Predicted**
GAVLK_NH_2	-4317334.10	-4315959.505	**y4**	**0.0095**	**1.0512**
GAVLK_NH_3	-4317321.17	-4315959.321	**y3**	**1.0000**	**1.0000**
GAVLK_NH_4	-4317318.06	-4315941.678	**y2**	**0.1405**	**0.0083**
GAVLK_NH_5	-4317328.14	-4315922.123	**y1**	**0.0451**	**0.00004**
